# Climate and Species Richness Predict the Phylogenetic Structure of African Mammal Communities

**DOI:** 10.1371/journal.pone.0121808

**Published:** 2015-04-15

**Authors:** Jason M. Kamilar, Lydia Beaudrot, Kaye E. Reed

**Affiliations:** 1 School of Human Evolution and Social Change, Arizona State University, Tempe, Arizona, United States of America; 2 Department of Anatomy, Arizona College of Osteopathic Medicine, Midwestern University, Glendale, Arizona, United States of America; 3 Graduate Group in Ecology, University of California Davis, Davis, California, United States of America; 4 Department of Anthropology, University of California Davis, Davis, California, United States of America; 5 Institute of Human Origins, Arizona State University, Tempe, Arizona, United States of America; Northern Illinois University, UNITED STATES

## Abstract

We have little knowledge of how climatic variation (and by proxy, habitat variation) influences the phylogenetic structure of tropical communities. Here, we quantified the phylogenetic structure of mammal communities in Africa to investigate how community structure varies with respect to climate and species richness variation across the continent. In addition, we investigated how phylogenetic patterns vary across carnivores, primates, and ungulates. We predicted that climate would differentially affect the structure of communities from different clades due to between-clade biological variation. We examined 203 communities using two metrics, the net relatedness (NRI) and nearest taxon (NTI) indices. We used simultaneous autoregressive models to predict community phylogenetic structure from climate variables and species richness. We found that most individual communities exhibited a phylogenetic structure consistent with a null model, but both climate and species richness significantly predicted variation in community phylogenetic metrics. Using NTI, species rich communities were composed of more distantly related taxa for all mammal communities, as well as for communities of carnivorans or ungulates. Temperature seasonality predicted the phylogenetic structure of mammal, carnivoran, and ungulate communities, and annual rainfall predicted primate community structure. Additional climate variables related to temperature and rainfall also predicted the phylogenetic structure of ungulate communities. We suggest that both past interspecific competition and habitat filtering have shaped variation in tropical mammal communities. The significant effect of climatic factors on community structure has important implications for the diversity of mammal communities given current models of future climate change.

## Introduction

A core goal of community ecology is to identify the processes that shape community structure, which refers to the richness and composition of species at a specific site and time. Assessing the relative importance of chance (stochastic processes) and ecological rules (deterministic processes) in shaping community structure is central to this goal [1–5]. Phylogenetic analyses have been used to quantify the structure of communities and help understand whether past interspecific competition or environmental filtering have played important roles in shaping community composition [6–10]. In particular, phylogenetic null model approaches can elucidate whether a community contains many closely or distantly related species relative to a random expectation from a larger assemblage of communities [6, 11–13]. If closely related species exhibit similar biological traits, and competition is strong among closely related species, then interspecific competition among closely related species that occupy similar niches may result in communities that are phylogenetically even (i.e. overdispersed). Alternatively, environmental filtering can result in communities that are phylogenetically clustered, which represents the co-occurrence of closely related species expected to share traits that are well suited for a given habitat.

While some research has examined the factors affecting the structure of tropical communities, much of this research has focused on plants (for review, see [14]). By comparison, we have limited understanding of tropical animal communities, and the processes that structure plant communities may or may not operate for faunal assemblages. Studies focusing on the phylogenetic structure of mammal communities have been relatively limited in terms of taxonomic and geographic sampling. Kamilar and Guidi [15] examined phylogenetic structure of primate communities and found that most communities were phylogenetically random, though Malagasy communities were phylogenetically even compared to those on other continents. Cardillo and Meijaard [16] focused on island mammal communities and also found that most exhibited a random phylogenetic structure, though there were some differences among taxonomic groups. In particular, many terrestrial ungulate communities on land-bridge islands showed significant levels of phylogenetic evenness. Another study examined a limited number of Neotropical monkey, possum, and squirrel communities and found a significant number of communities that were phylogenetically even at the Class level, though only the monkey and squirrel communities showed a similar pattern at the Order level [17].

In recent years, some studies have more explicitly investigated how environmental variation affects the phylogenetic structure of communities. For example, Graham and colleagues [13, 18] clearly demonstrated that community phylogenetic metrics (e.g. net relatedness index and nearest taxon index) are informative and biologically meaningful even in cases where many communities exhibit a phylogenetic structure that does not significantly differ from the null expectation. Both of these studies found that the phylogenetic structure of hummingbird communities varied along environmental gradients. In particular, hummingbird assemblages were phylogenetically clustered in high elevation sites and seasonally dry lowlands. In contrast, communities were more phylogenetically even in wet lowlands. This suggests that closely related species with similar biological characteristics likely have some adaptations to more challenging environments, such as those found at high elevations and seasonally dry sites.

Although previous studies have generally found that most mammal communities exhibit a phylogenetic structure that is not statistically different from random, new research has found that the phylogenetic structure of some mammal communities does vary in response to environmental factors. Cardillo [19] used 102 terrestrial ecoregions as his sampling units (as opposed to study sites, parks, etc.) in a study focused on the phylogenetic structure of African carnivore assemblages. He also grouped the ecoregions into biomes, which essentially reflect different habitat types (e.g. tropical and subtropical moist broad leaf forests, deserts and xeric shrublands, etc.). Although a direct test of the relationship between habitat type and community structure was not performed, there was variation in the phylogenetic structure of carnivore assemblages across biomes. In addition, a recent paper by Cantalapiedra et al. [20] found that a global distribution of ruminant assemblages were most phylogenetically clustered in three relatively extreme climate domains, the Sahara Desert, the Arabian Desert, and southwestern Arabian savannas. They argue that this may be evidence that ruminants living in these environments require specialized adaptations. It is interesting to note that they did not find significant phylogenetic clustering of ruminant assemblages in other habitats that may be considered extreme, such as cold deserts or tundra.

Our knowledge of how climatic variation influences the phylogenetic structure of vertebrate communities and whether these effects are consistent across taxonomic groups is still quite limited. Thus, the goal of this paper is to quantify the phylogenetic structure of mammal communities in Africa and more importantly, to examine how this structure varies in response to the significant climatic variation across the continent. In addition, we test the stress gradient hypothesis [21], which suggests that competitive interactions are stronger where conditions are less harsh. We expect environmental filtering to result in clustering in harsh environments and competition to result in overdispersion in tropical environments where resources are abundant. Therefore, we predict that mammal communities will be more phylogenetically clustered (i.e. contain more closely related species) in harsher environments, i.e. habitats with overall low rainfall, and high rain and/or temperature seasonality. Coping with harsh environments likely requires specific biological traits that are likely to be shared among closely related taxa. In contrast, phylogenetically even communities (i.e. communities with a relatively high proportion of distantly related species) are likely more common in high quality habitats. Overdispersion may be due to past interspecific competition and competitive exclusion among closely related species, resulting in modern communities comprised of more distantly related taxa. In addition, these habitats are more complex in terms of vertical stratification and in the number of plant species, which provide the context for a wide range of mammalian niches [22, 23]. If distantly related species occupy disparate niches, then this will likely result in phylogenetically even communities. Examining this question in Africa is well suited to the goals of comparing community structure with climatic influences because of the diverse habitats found across the continent. Relatively dry, xeric habitats are found in discontinuous distributions in the south, east, and northern portions of the continent. This provides a test of whether similar habitats produce convergent community structure. Finally, we expect that communities with higher species richness will exhibit more phylogenetically even structures. This pattern was found in a recent analysis of African haplorhine primates [9]. The authors argued that increased interspecific competition in larger communities may produce this result. Alternatively, larger communities are typically found in more productive and diverse habitats (e.g. rainforests) that may contain a wider variety of niches and subsequently more diverse mammal taxa.

In addition, important biological differences among mammal orders related to their trophic level may have implications for variation in community structure and macroecology across orders [24–27]. For instance, dietary differences among orders may influence how communities vary with respect to environmental variation. African ungulates rely solely on a plant diet, whereas invertebrates and vertebrates comprise an important part of many primate and carnivoran species diets, respectively [28–30]. Therefore, we expect that ungulate community structure will be more affected by environmental variation, because environmental factors likely shape plant abundance and diversity more directly than influencing invertebrate and vertebrate prey species.

## Methods

### Data Collection

We compiled mammal lists from information on African national parks, game reserves, and protected areas, rather than extracting assumed presence/absence data from species range maps, which often overestimate presences [31, 32]. For each site, we recorded species presence-absence data (i.e. species lists for each mammal community) from a variety of sources, including: 1) existing species locality databases [15, 33], 2) published research articles that examined mammal distributions and/or communities, and 3) published field surveys. All mammal Orders were included in our database except Chiroptera. A species within an Order was only included if it had a body mass of greater than 500 grams because reliable presence-absence data for smaller mammals often requires different sampling effort. Also, we conducted additional analyses on the three most species rich clades in our dataset: Primates, Carnivora and terrestrial Artiodactyla/Perrisodactyla (hereafter called ungulates). Our total dataset contains 243 mammal species, of which 57 are primates, 69 are carnivorans and 89 are ungulates at 203 sites across Africa (S1 Dataset). While we acknowledge that some regions within Africa are better sampled than others, our dataset contains representative communities from all major environments/habitats within the continent. Therefore, our tests examining the climatic predictors of community phylogenetic structure should be robust.

Each locality was recorded with centralized geospatial coordinates (i.e., the center of the site’s latitude and longitude). We also associated each site with high-resolution climate data from the WorldClim database [34], which has been extensively used in macroecological research of mammals and other vertebrates [18, 35–37]. In particular, we used six of the 19 “bioclim” variables available from the database, as these variables well characterized the local climate and were not highly correlated. These variables included: 1) annual mean temperature, 2) temperature seasonality, 3) minimum temperature of coldest month, 4) annual precipitation, 5) precipitation of driest month, and 6) precipitation seasonality. In addition to quantifying the abiotic environment, these variables serve as proxies of habitat structure [35, 38, 39].

### Data Analysis

Most commonly, community phylogenetic structure has been quantified using two metrics, the net relatedness index (NRI) and the nearest taxon index (NTI) [6, 40]. The former is a measure of the mean phylogenetic distance among all species in a community relative to that found in the species pool (i.e. all possible species found in all communities in the dataset). The latter metric quantifies the phylogenetic distance among the most closely related species in a community, relative to the species pool. Thus, NTI measures the phylogenetic distance between species at the tips of the phylogeny. Assuming that closely related species exhibit similar biological characteristics, significantly low net relatedness index (NRI) and nearest taxon index (NTI) values (i.e. phylogenetic evenness) can indicate past interspecific competition, resulting in closely related species not found in the same community. Alternatively, significantly high NRI/NTI values (i.e. phylogenetic clustering) suggest that environmental filtering is important for influencing community composition because closely related species tend to have similar ecological requirements. Because NRI and NTI are measuring two different aspects of community phylogenetic structure, it is possible that they produce somewhat different results. Importantly, NTI is likely better to capture interspecific competition because it focuses on the most closely related species in a community, whereas NRI are more likely to detect the effects of environmental filtering [41]. Importantly, NTI values may reflect possible interspecific competition because there is good evidence supporting the idea that closely related (e.g. within a genus) mammal species exhibit similar biological characteristics [42–44]. Communities may also exhibit phylogenetically random species compositions, yielding NRI/NTI scores not significantly different from zero [40]. NRI and NTI scores are also informative and biologically relevant even if they do not significantly differ from zero [18]. For example, a community with a NRI value of -1.5 contains more distantly related species than a community was a value of -0.5, even if neither community yields a statistically significant p value.

These metrics were quantified with the PHYLOCOM software package [40]. We used 4999 randomizations to calculate statistical significance. We employed the independent swap null model [40, 45, 46]. This null model has important advantages over other models. In particular, phylogenetic structure is not influenced by phylogenetic signal in species prevalence [47].

We used the mammal supertree presented in Bininda-Emonds et al. [48, 49] as the basis for our phylogeny because it contains all of the taxa in our dataset and is regularly used in broad scale comparative analyses of mammals [19, 50, 51]. There were some cases where “old” data sources reported a taxonomic name that was not found in Bininda-Emonds et al. [48,49]. In these cases we synonymized the species in the original data source to the Bininda-Emonds et al. [48,49] phylogeny. We modified the primate portion of the supertree by using a primate consensus tree obtained from the 10K Trees Project Version 3 [52]. This primate phylogeny was an improvement because it contained many nodes that were better resolved (i.e. contained fewer polytomies) than those found in the Bininda-Emonds et al. [48, 49] tree. When incorporating the primate portion of the phylogeny with the remaining mammals, we increased the branch length leading to primates by ~18 my to insure that the tips of the tree were contemporaneous.

We utilized Kruskal-Wallis tests with Monte Carlo simulation to test for differences in the NRI and NTI values among the three clades of mammals for which we obtained the most data: carnivores, primates, and terrestrial ungulates. We used 9999 randomizations to generate p values. We conducted these analyses using the coin package [53] for R [54].

We used simultaneous autoregressive models (SAR) [55] to test the effects of species richness and climate for predicting our measures of community phylogenetic structure. This method is advantageous over a typical linear regression because it accounts for spatial autocorrelation in the residuals of the model. All variables were log transformed prior to analysis.

We calculated NRI and NTI values for our total mammal dataset (i.e. all mammals in the Orders found in our 203 communities), as well as for three mammal subsets: primate communities (135), carnivoran communities (199), and ungulate communities (183) that we analyzed separately. At least four species were required in a community for the site to be included in the analysis. In addition, we conducted separate SAR analyses for each of the aforementioned datasets. We used the Spatial Analysis in Macroecology software package [56] to conduct all SAR analyses.

## Results

**Fig 1:**
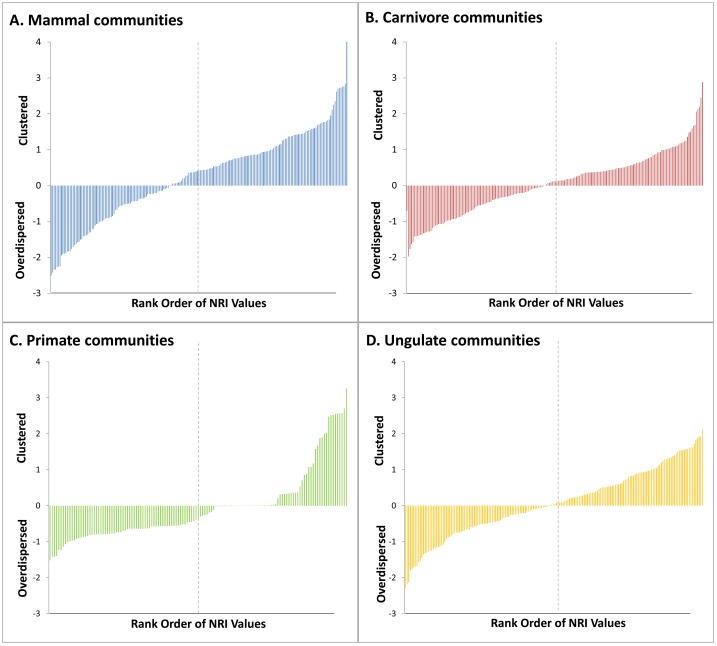
Rank order of Nearest Taxon Index values for the four community datasets examined: A) All mammals, B) Carnivores only, C) Primates only, D) Ungulates only. Vertical dashed lines indicates 50^th^ percentile.

**Fig 2:**
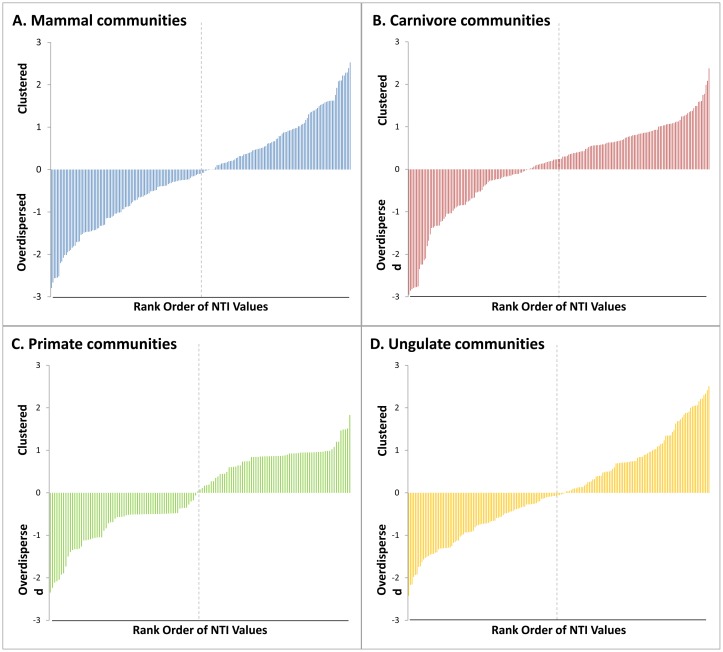
Rank order of Net Relatedness Index values for the four community datasets examined: A) All mammals, B) Carnivores only, C) Primates only, D) Ungulates only. Vertical dashed lines indicates 50^th^ percentile.

Results for all groupings are presented in Table 1 and visualized in Figs. 1 and 2. When using the NRI metric, we found that 89.7% (182) of the 203 mammal communities exhibited a random phylogenetic structure, 5.9% (12) were significantly phylogenetically even, and 5.4% (11) were significantly clustered. When using the NTI metric, 90.2% of mammal communities were randomly structured, 5.4% (11) were significantly phylogenetically even, and 4.4% (9) were significantly clustered (Table 1 and Figs. 1 and 2).

**Table 1:** The phylogenetic structure of African mammal communities. The number of communities followed by the frequency and percentage of significantly structured communities are specified.

Dataset	# Communities	# Low NRI	% Low NRI	# High NRI	% High NRI	# Low NTI	% Low NTI	# High NTI	% High NTI
All mammals	203	12	5.91	11	5.42	11	5.42	9	4.43
Primates	135	4	2.96	3	2.22	5	3.70	1	0.74
Carnivora	198	0	0.00	4	2.02	8	4.04	5	2.53
Ungulates	182	3	1.65	7	3.85	6	3.30	13	7.14

NRI = Net relatedness index. NTI = Nearest taxon index.

These patterns differed when examining finer taxonomic scales individually. At this level of analysis, a greater proportion of communities exhibited a random phylogenetic structure based on the NRI metric. Specifically, 94.8% (128) of primate, 98.0% (195) of carnivoran, and 94.5% (173) of ungulate communities had a random phylogenetic structure. Significant phylogenetic evenness was observed in 3.0% (4) of primate, 0% of carnivoran, and 1.7% (3) of ungulate communities, while phylogenetic clustering accounted for 2.2% (3) of primate, 2.0% (4) of carnivoran, and 3.9% (7) of ungulate communities. In contrast, a lower proportion of communities were phylogenetically random using the NTI metric, especially for carnivores and ungulates. A random phylogenetic structure was found in 95.6% (129) of primate, 93.7% (186) of carnivore, and 89.6% (164) of ungulate communities. A statistically significant degree of phylogenetic evenness was observed for 3.7% (5) of primate, 4.0% (8) of carnivore, and 3.3% (6) of ungulate communities. Phylogenetically clustered patterns were present in 0.7% (1) of primate, 2.5% (5) of carnivore and 7.1% (13) of ungulate communities.

**Fig 3:**
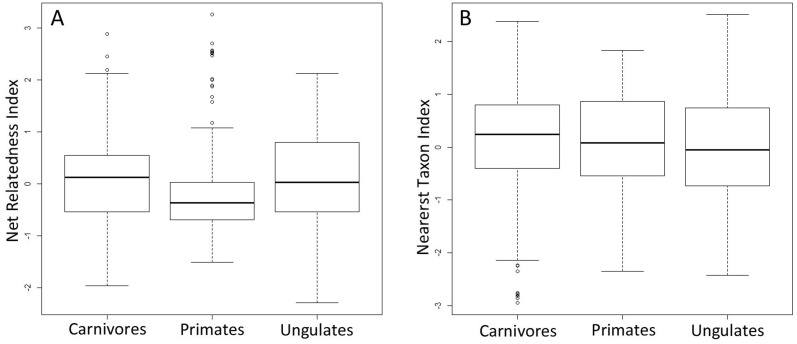
Boxplot of A) Net Relatedness Index and B) Nearest Taxon Index values of communities across mammal clades.

The results of our Kruskal-Wallis tests with Monte Carlo simulation yielded a statistically significant difference in NRI values across mammal clades (p = 0.012) (Fig. 3A). Primate communities, on average, exhibited lower values than carnivores and ungulates. In contrast, NTI values did not significantly differ across mammal groups (p = 0.448) (Fig. 3B).

**Fig 4:**
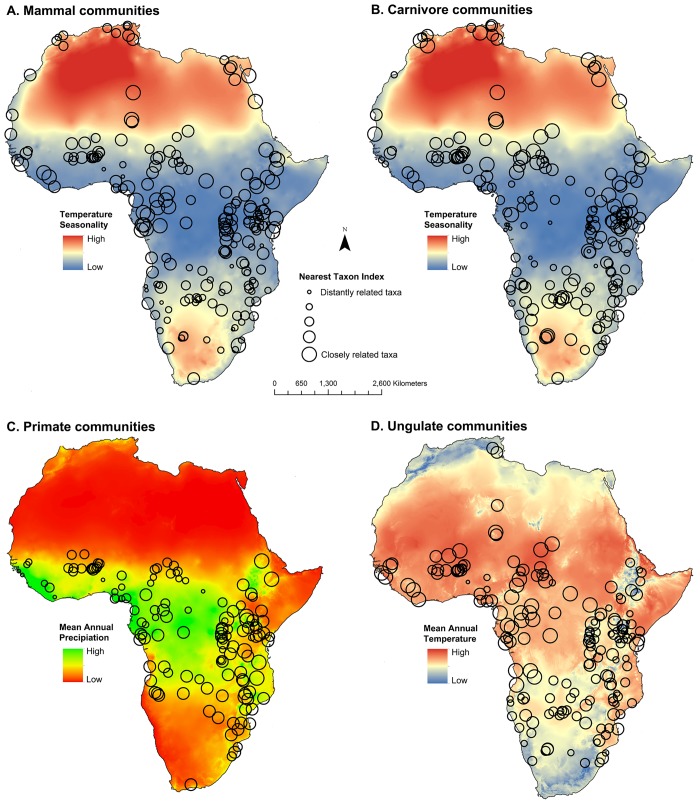
The relationship between climatic factors and the phylogenetic structure of mammal, carnivore, primate, and ungulate communities based on the Nearest Taxon Index.

**Fig 5:**
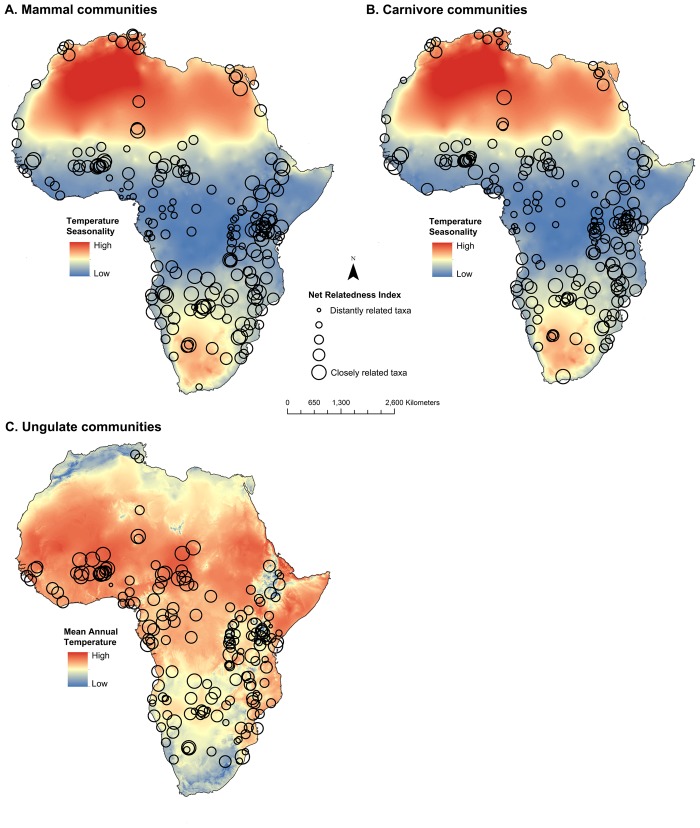
The relationship between climatic factors and the phylogenetic structure of mammal, carnivore, primate, and ungulate communities based on the Net Relatedness Index.

Using simultaneous autoregressive models, the phylogenetic structure of mammal communities was significantly predicted by community species richness and climatic variables, though the relationship between these variables and community structure varied across taxonomic scale and across Orders (see Tables 2 and 3 and Figs. 4 and 5). Two variables, species richness and temperature seasonality, best predicted the NTI phylogenetic structure of mammal communities. As community size and temperature seasonality increased, mammal communities became increasingly phylogenetically even (i.e. more negative NTI values). These two variables also predicted carnivoran community structure, yet temperature seasonality was related in the opposite direction. Increases in community size and decreases in temperature seasonality resulted in increasing phylogenetic evenness in carnivoran communities. For primate communities, only one variable, mean annual rainfall was a significant negative predictor of NTI values, thus communities in high mean annual rainfall were more phylogenetically even and communities in dry sites were phylogenetically clustered. The NTI values of ungulate communities were best predicted by five variables. Community species richness (-), annual mean temperature (+), temperature seasonality (-), minimum temperature of coldest month (-), and annual rainfall (+), were all significant predictors of the NTI values of ungulate communities.

**Table 2:** Results of simultaneous autoregressive models predicting the nearest taxon index of mammal communities from species richness and climate data.

	All Mammals		Carnivora		Primates		Ungulates	
Predictors	Std. Coeff.	p value	Std. Coeff.	p value	Std. Coeff.	p value	Std. Coeff.	p value
Species richness	**-0.374**	**<0.001**	**-0.198**	**0.009**	-0.194	0.072	**-0.459**	**<0.001**
Ann. mean temp	0.104	0.519	0.183	0.255	-0.095	0.663	**0.539**	**<0.001**
Temp. seasonality	**-0.453**	**0.004**	**0.331**	**0.039**	-0.109	0.521	**-0.384**	**0.008**
Min. temp. coldest month	-0.049	0.796	-0.194	0.316	-0.080	0.700	**-0.495**	**0.005**
Ann. precipitation	0.053	0.589	-0.079	0.382	**-0.252**	**0.026**	**0.253**	**0.005**
Precip. of driest month	-0.040	0.742	0.144	0.244	0.202	0.197	0.060	0.611
Precip. seasonality	-0.116	0.295	0.146	0.199	0.167	0.279	0.015	0.895
Total Model Results	F = 5.892, r² = 0.309, p <0.001, n = 203		F = 7.241, r² = 0.275, p<0.001, n = 198		F = 4.931, r² = 0.285, p<0.001, n = 135		F = 13.294, r² = 0.435, p<0.001, n = 182	

All variables were log transformed prior to analysis.

**Table 3:** Results of simultaneous autoregressive models predicting the net relatedness index of mammal communities from species richness and climate data.

	All Mammals		Carnivora		Primates		Ungulates	
Predictors	Std. Coeff.	p value	Std. Coeff.	p value	Std. Coeff.	p value	Std. Coeff.	p value
Species richness	0.155	0.070	-0.031	0.699	**-0.257**	**0.030**	**-0.364**	**<0.001**
Ann. mean temp	0.132	0.366	0.031	0.857	0.184	0.445	**0.472**	**0.006**
Temp. seasonality	**0.341**	**0.017**	**0.379**	**0.029**	-0.322	0.087	-0.187	0.251
Min. temp. coldest month	-0.136	0.425	0.096	0.646	-0.055	0.810	-0.375	0.060
Ann. precipitation	-0.120	0.170	**0.228**	**0.020**	0.047	0.704	0.190	0.058
Precip. of driest month	-0.112	0.310	-0.045	0.734	0.117	0.493	-0.038	0.778
Precip. seasonality	0.034	0.732	-0.05	0.684	0.066	0.696	0.068	0.602
Total Model Results	F = 9.801, r² = 0.355, p <0.001, n = 203		F = 1.424, r² = 0.142, p = 0.198; n = 198		F = 2.201, r² = 0.254, p = 0.038, n = 135		F = 7.773, r² = 0.274, p<0.001, n = 182	

All variables were log transformed prior to analysis.

The models predicting the phylogenetic structure of communities based on NRI values produced different results (Table 3). First, the total mammal dataset was best predicted by temperature seasonality, though in the opposite direction of NTI. In this model, increasing temperature seasonality resulted in mammal communities that were phylogenetically clustered. For carnivoran communities, temperature seasonality and mean annual rainfall were positively related to NRI values (i.e. phylogenetic clustering). The NRI values of primate communities were significantly predicted by species richness only (p = 0.030). Finally, species richness (-) and annual mean temperature (+) were the strongest predictors of ungulate community NRI values. Additional detailed results are presented in S1 Dataset.

## Discussion

We found a strong effect of both local climate (and therefore, habitat) and species richness on the phylogenetic structure of mammal communities. This supports the idea that African mammal communities have converged on a similar phylogenetic structure when found in similar climatic contexts, even when these communities are found in geographically distinct parts of the continent. In addition, the specific climatic factors influencing community structure varied across mammalian clades, suggesting that differences in the biological traits of these orders plays an important role in their community ecology and evolution, specifically dietary niche. Ungulates are solely primary consumers, whereas other clades, such as carnivorans and primates, comprise species that consume both plant and animal matter [30, 42, 57, 58].

### The Phylogenetic Structure of Individual Communities

Most individual communities did not exhibit significant phylogenetic structure. This supports prior studies examining mammal community structure in other regions of the world, as well as studies using smaller African datasets [15, 17, 59]. Interestingly, the proportion of mammal communities that were significantly phylogenetically structured depended on the dataset. For instance, including all mammal species resulted in the highest proportion of both significantly low and high net relatedness index (NRI) values and low nearest taxon index (NTI) values. Terrestrial ungulate communities yielded the highest proportion of significantly high NTI values, indicating that communities contained species that were closely related relative to the null model. Further differences in the phylogenetic community structure of our three mammal clades of interest were detected, for the NRI metric in particular. Primate communities had lower NRI values compared to carnivorans and ungulates. NRI values are based on the phylogenetic distance among all members of a community and lower values indicate that members of primate communities are more distantly related than other mammal clades. This is likely due to the fact that many primate communities in the analysis contained multiple species from the primate suborders Strepsirrhini and Haplorrhini, which diverged about ~70mya [48, 49]. In contrast, many carnivoran communities are comprised of several species from the families Felidae, Herpestidae, and Viverridae (which diverged at ~40mya), but only one or few representatives of the Canidae (which diverged from the aforementioned families at ~60mya). Therefore, many primate communities often contain more distantly related species compared to other mammal communities. These results strongly suggest that a clade’s evolutionary history and pattern of diversification can have an important impact on the phylogenetic structure of modern communities.

An important assumption of the community phylogenetic framework is the idea that species traits relevant for interspecific competition have strong phylogenetic signal. If this assumption does not hold (e.g. when there are numerous instance of convergent evolution among distantly related species), then competition may actually be stronger between distantly related species, rather than closely related ones. In addition, some traits may exhibit weak phylogenetic signal, being randomly distributed across the phylogeny [42]. If true, then this may help to explain the relatively high proportion of communities with a phylogenetic structure no different from a null expectation. In addition, other confounding factors, such as shared predators, may produce similar patterns expected under an interspecific competition rationale.

### Species Richness and the Phylogenetic Structure of Communities

Species richness was a consistently negative predictor of the net relatedness index (NRI) of communities, i.e. increasingly large communities were associated with more distantly related species. These results are similar to those found in a recent study focused solely on African haplorhine primate communities [9]. One interpretation is that interspecific competition among closely related species in the past may have resulted in more distantly related congeners in species rich communities, particularly if past interspecific competition has resulted in niche differentiation and resource specialization. Communities with the greatest numbers of species occur in the Congo Basin and nearby regions. Rainforest habitats likely support numerous herbivorous and omnivorous species because of relatively consistent availability of high plant and insect biomass and diversity [60, 61], which can in turn support numerous carnivorous species (preying on vertebrates). The complex forest structure allows for both horizontal and vertical stratification of resources, including potential dietary items, sleeping sites, etc. [22]. Additionally, rainforests have likely had the least contact from humans, which may have influenced lower diversity in higher contact regions [62]. These results also support the stress gradient hypothesis [21], with high productivity environments (as defined by high species richness only) being associated with phylogenetically even communities.

The relationship between species richness and community phylogenetic structure may be consistent across a wide array of organisms. This same pattern was found in a recent study by Qian et al. [63] that examined North American angiosperm trees. Interestingly, their analyses treated community structure as the predictor variable and species richness as the dependent variable. The authors argue that this is evidence for species richness being driven by evolutionary time and niche conservatism. Considering our work and the study by Qian et al. [63], we suggest that the causal direction of the relationship between richness and community structure requires further investigation.

### Climate and the Phylogenetic Structure of Communities

The importance of climate for predicting community structure varied across mammalian clades. When all mammals were considered together, temperature seasonality was negatively related to NTI values, indicating that more seasonal sites contained communities with relatively distantly related species. Conversely, mammal communities contained more closely related species in sites with low levels of temperature seasonality. This result suggests that closely related mammal species may be unable to coexist in seasonal habitats due to limited resources, which may have resulted in competitive exclusion in these areas. Alternatively, an evolutionary explanation may be rooted in the idea that taxa inhabiting seasonal habitats may have lower speciation rates than those in regions with higher productivity and climate stability (e.g. equatorial rainforests) [63, 64]. In addition, this finding does not support the stress gradient hypothesis [21], which predicts that competitive interactions will be stronger in less harsh, less seasonal environments. Phylogenetic overdispersion/evenness in sites with high temperature seasonality contradicts the support for the stress gradient hypothesis based on the results of species richness and community NTI values in which phylogenetic overdispersion was higher in species rich communities. Since the models examined all predictor variables simultaneously, the temperature seasonality effect on community structure is independent of species richness and thus the combination of these results can be considered as mixed support for the stress gradient hypothesis when all mammals in this study were considered.

Temperature seasonality was the most consistent predictor of community phylogenetic structure, yet the direction of the relationship differed across orders. Increased temperature seasonality resulted in more phylogenetically even communities across all mammals and within ungulates. Terrestrial ungulate communities were best predicted by environmental variables when using the NTI metric (and generally similar results when using NRI). This supports the idea that ungulate communities may be more affected by environmental factors as primary consumers, compared to primates and carnivorans, which consume both invertebrate and vertebrate prey. The herbivorous ungulates divide their diets into grazers, browsers, and mixed feeders, with browsers also sometimes consuming fruit and mixed feeders usually preferring grass or leaves while eating the other seasonally [65–67]. The biomass of their preferred plant food sources may be reduced in areas with increased temperature seasonality, suggesting that the species in the communities handle food shortages with differing functional traits. This may restrict the number of closely related species that can live in the same community, as they tend to have the same dietary strategies [68].

In contrast, increased temperature seasonality was related to more phylogenetically clustered carnivoran communities based on both NTI and NRI values. Carnivoran species living in more seasonal habitats may require specific adaptations, which are common in closely related species, to either cope with the seasonal variation in temperature itself, or to manage the challenges in acquiring proper nutrition.

Interestingly, there was only a single predictor of NTI variation in primate communities, mean annual precipitation. As precipitation increased, primate communities contained more distantly related species. Primates are typically regarded as forest adapted mammals [29] and our results quantitatively confirm this idea that the most phylogenetically diverse primate assemblages are found in rainforest sites. This finding suggests that past interspecific competition may have driven the absence of closely related taxa in rainforest habitats. An alternative explanation (but not mutually exclusive) relies on the idea that rainforests contain a diverse array of niches [22]. If distantly related primate species occupy distinct niches, then we would expect phylogenetically diverse communities in these habitats.

### Mammal Communities, Species Traits, and Climate Change

Explaining the patterns of community phylogenetic structure relies somewhat on the assumption that closely related species have similar biological characteristics (and therefore, occupy similar niches) and this similarity declines with increasing phylogenetic distance. In fact, a recent study by Kamilar and Cooper [42] showed that a wide array of primate traits exhibited a significant amount of phylogenetic signal. This finding supports the use of phylogeny as a surrogate for biological similarity. To our knowledge, similar studies of carnivorans and ungulates have not been performed. Future studies quantifying the degree of phylogenetic signal in traits for these clades can further refine our predictions of community phylogenetic structure. If functional traits exhibit weak phylogenetic signal, there is less of an expectation that communities will be phylogenetically clustered or even. Also, future studies may provide more detailed information about the mechanisms driving community structure by examining species traits in a community context [69, 70]. In particular, investigating the relationship between the phylogenetic structure of communities, functional trait variation and the degree of phylogenetic signal in these traits has recently begun [8, 18, 69] and is an important future direction.

Finally, our results have important implications for future mammal diversity, especially at the community level. Many of the communities in our dataset are found in the tropics, which are among the most diverse ecosystems in the world. These areas are among the world’s biodiversity hotspots, which are critically important for conservation [71, 72], and contribute to climate stability [73]. Models predicting future climate change show noticeable shifts in temperature and rainfall patterns, especially if current anthropogenic impacts continue unabated [74]. The significant relationships we found between the phylogenetic structure of mammal communities and climatic variation strongly suggest that future climate change will result in altered mammal community structure.

## Supporting Information

S1 Dataset Data used in analyses and additional results. (XLSX)
